# Machine learning for predicting acute hypotension: A systematic review

**DOI:** 10.3389/fcvm.2022.937637

**Published:** 2022-08-23

**Authors:** Anxing Zhao, Mohamed Elgendi, Carlo Menon

**Affiliations:** ^1^Biomedical and Mobile Health Technology Lab, ETH Zürich, Zürich, Switzerland; ^2^Department of Physics, ETH Zürich, Zürich, Switzerland

**Keywords:** digital health, hypotension, hypertension, intensive care unit, anesthesia, obstetric and gynecologic, emergency and critical care, low blood pressure

## Abstract

An acute hypotensive episode (AHE) can lead to severe consequences and complications that threaten patients' lives within a short period of time. How to accurately and non-invasively predict AHE in advance has become a hot clinical topic that has attracted a lot of attention in the medical and engineering communities. In the last 20 years, with rapid advancements in machine learning methodology, this topic has been viewed from a different perspective. This review paper examines studies published from 2008 to 2021 that evaluated the performance of various machine learning algorithms developed to predict AHE. A total of 437 articles were found in four databases that were searched, and 35 full-text articles were included in this review. Fourteen machine learning algorithms were assessed in these 35 articles; the Support Vector Machine algorithm was studied in 12 articles, followed by Logistic Regression (six articles) and Artificial Neural Network (six articles). The accuracy of the algorithms ranged from 70 to 96%. The size of the study sample varied from small (12 subjects) to very large (3,825 subjects). Recommendations for future work are also discussed in this review.

## Introduction

It is widely accepted that hypotension is defined as absolute mean arterial pressure (MAP) below 60–65 mmHg (1). The incidence of hypotension is estimated to affect around half of the population worldwide (2, 3). While chronic low blood pressure without symptoms is usually not concerning, health problems may occur when blood pressure drops suddenly.

An acute hypotensive episodes (AHE) is defined as lasting for 30 min to 1 h or longer during which at least 90% of the MAP is at or below 60 mmHg. While this definition is widely used, it is not based on consensus or is not part of a medical guideline; rather, it is from the 10th PhysioNet/CinC Challenge (2009) (4). It has also been noted that intra-operative hypotension should be defined as a relative difference from baseline MAP (5, 6). AHE often happens in the intensive care unit (ICU) or operation rooms, commonly caused by sepsis, myocardial infarction, cardiac arrhythmia, pulmonary embolism, hemorrhage, dehydration, and anaphylaxis (4). Hypotension reduces the oxygen supply, resulting in cell and tissue injury and loss of function. Therefore, AHE requires an immediate and appropriate intervention. Without this, patients are at an increased risk of irreversible organ damage and even death.

Currently, several scoring systems are used to predict critical medical events; however, these systems have not been specifically developed for AHE (7, 8). The symptoms of AHE may not be noticeable, and they might last only a few seconds. Hence, an adequately early prediction or warning system is desired to give nurses and physicians enough time to administer preventive care. This is especially important in an ICU setting, as often there is a shortage of nurses.

The importance of predicting AHE was first noted in the European AVERT-IT (Advanced Arterial Hypotension Adverse Event prediction through a Novel Bayesian Neural Network) project in 2008, which was funded by the European Commission to develop a novel bedside monitoring and alerting system to predict AHE (9). In 2009, in the 10th PhysioNet/CinC Challenge, using an automated method, the participants were expected to predict which patients in the challenge dataset (MIMIC II) would experience an AHE (4). Since the challenge, there has been continuous interest in this topic, and more researchers have studied it.

This paper reviews the relevant literature published between 2008 and 2021. Before 2008, there were very few studies in this area, and the methodologies were mainly statistical models rather than machine learning algorithms. Given the advances in machine learning in the last decade, we are re-visiting this topic with a focus on answering the following questions: (1) How well do machine learning algorithms perform in predicting AHE? and (2) What is the potential of these current ML algorithms in the clinical setting?

## Methods

###  Study guidelines

This review was conducted according to the Preferred Reporting Items for Systematic Reviews and Meta-Analyses statement (PRISMA) (10). A prior review protocol was drafted using the Preferred Reporting Items for Systematic Reviews and Meta-Analyses Protocols (11) for internal use amongst the research team but it was not externally published or registered prospectively.

###  Search strategy and study eligibility

The PubMed, IEEE database, Embase, and Google Scholar were searched for articles published between January 1, 2008, and January 1, 2022, for all English-language papers using the following keywords: (hypotensive or hypotension or low blood pressure) and (ECG or electrocardiogram or MIMIC) and (automatic detection or machine learning or artificial intelligence or deep learning or prediction) were used. The detailed strategy was discussed in Supplementary material. Gray literature was not included in this review in an attempt to only include peer-reviewed studies. This timeframe was chosen to reflect advances in artificial intelligence technologies and applications in medicine. The search for this review was completed in May 2022.

### Inclusion and exclusion criteria

Articles were excluded (a) if the focus was not on hypotension, (b) if ECG data were not used, (c) if a machine learning algorithm was not used, (d) if the article was a review, a book chapter, or a thesis, and (e) if the article did not address the topic (predicting hypotension). One reviewer (AZ) conducted the literature search and two reviewers (AZ and ME) screened the titles, abstracts and full-texts independently for potentially eligible studies. Reference lists of eligible studies were also hand-searched but no additional studies were included on this basis.

###  Study selection and data extraction

One author (AZ) conducted the literature search, and two authors (AZ and ME) independently screened the titles and abstracts for potentially eligible studies. Each potential study for inclusion underwent full-text screening and was assessed to extract study-specific information and data. For each of the included articles, we extracted information from the below perspectives: the year the paper was published, author(s), number of subjects, gender split of the subjects, the signal used, sampling frequency, features extracted, machine learning algorithms evaluated in the study, training data window length, prediction window length, data source, evaluation metric(s) of the machine learning algorithms.

## Results

### Search results

**Figure 1:**
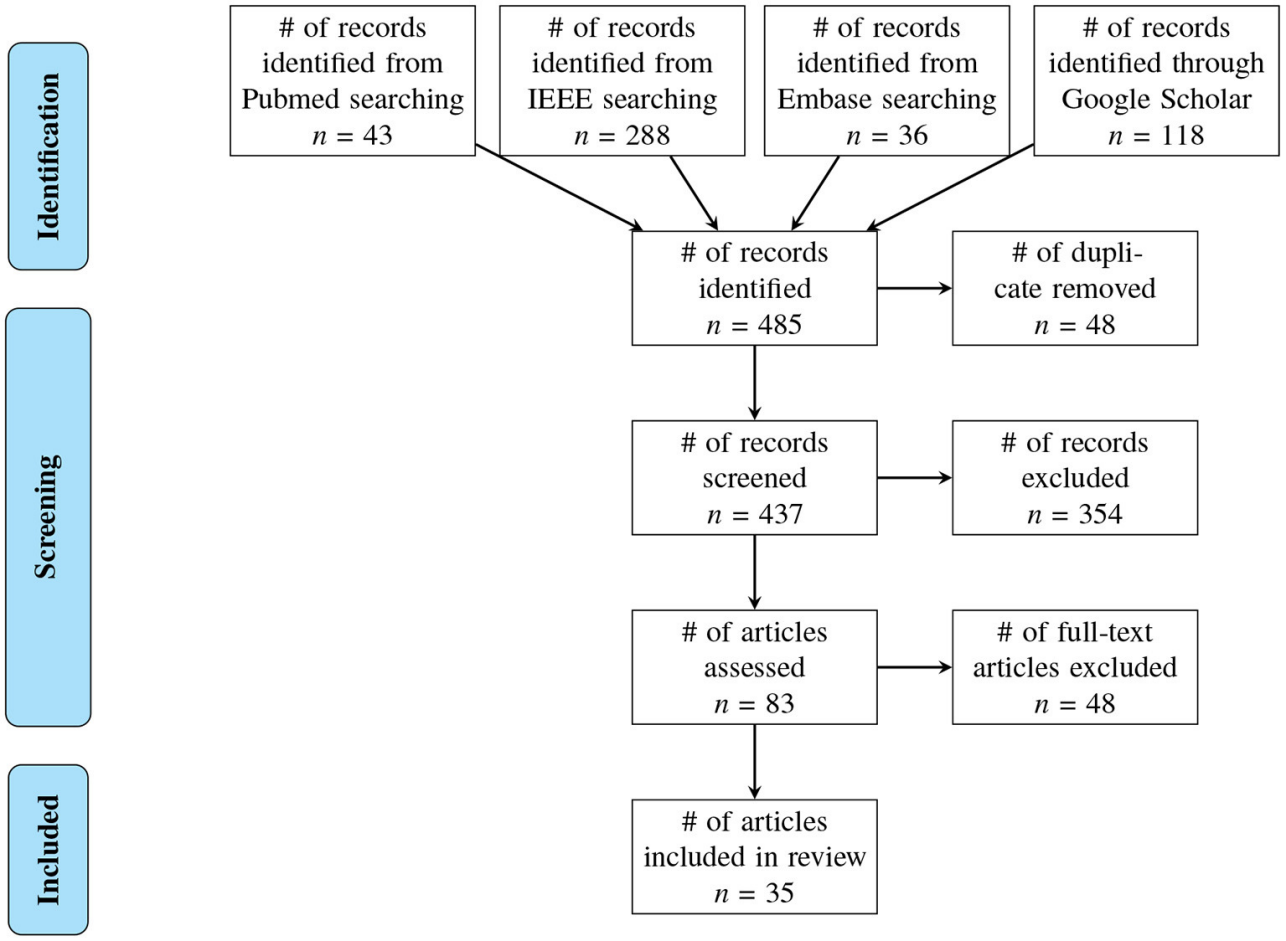
Flow chart of the methodology used to screen the articles. Thirty-five articles published between 2008 and 2021 were included in the review.

As shown in Figure 1, a total of 485 records were identified with the above-mentioned keywords and year range in the four databases. After comparing the literature titles and authors, 48 duplicates were confirmed and removed, resulted in 437 search records. With the five exclusion criteria mentioned in the Methods section, 354 of the 437 records were excluded after reading the abstract: 40 studies did not use electrocardiogram (ECG) as one of the signals; 161 studies did not investigate hypotension; 63 studies did not aim for blood pressure prediction; 89 were a review article or chapter in a book or a thesis; fiver were excluded because they were either not written in English or the full text was unavailable.

The full text was assessed in 83 articles. Of those, 48 articles were further excluded: 11 studies did not use machine learning as a forecasting method; 16 studies focused on blood pressure estimation rather than prediction; six studies forecasted blood pressure in general but not as a way to predict AHE; six studies used blood pressure to forecast other diseases; three studies aimed to determine which feature has the greatest predictive power, rather than to assess an algorithm; one study used an animal model, one focused on developing a new sensor, one aimed to detect artifacts, and one focused on photoplethysmography rather than ECG. Ultimately, 35 articles were included in this systematic review.

### Characteristics of included reviews

We read and summarized the included articles based on (a) the year in which the article was published, (b) how many subjects were included in the study and the gender info, (c) what signal(s) was/were used, and the sampling frequency used to obtain the signal, (d) what features were extracted, (e) what machine learning algorithm(s) was/were evaluated in the study, (f) the evaluation metrics of the machine learning algorithm, (g) the duration of the observation window and prediction window, and (h) what data source the authors referred to, as shown in Table 1.

**Table 1:** Overview of studies included in the systematic review.

**References**	**Subject**	**Gender split**	**Signal**	**Samp Freq**	**Feature**	**ML algorithm**	**Training window**	**Predicted length**	**Data source**	**Evaluation metric**
Zhang et al. (12)	1,055	*F* = 425	ABP	N/R	Max, min, avg, median, STD, skewness, kurtosis, upper quartile, avg absolute deviation, range, variance	•LR •AdaBoost •SVM •RF •XGBoost •Gradient boosting •Ensemble	5 h	60 min	MIMIC II (13)	Accuracy(%) •LR = 77.8 •AdaBoost = 82.0 •SVM = 80.1 •RF = 81.0 •XGB = 80.4 •GB = 81.0 •Ensemble = 82.2
Ribeiro et al. (14)	3,825	N/R	HR, RR, SpO2, SBP, DBP, MAP time series, PP, CO	N/R	Interquartile range, max, min, mean, median, skewness, kurtosis, linear slope, SD, variance, wavelet energy, cross-correlations between signals	Layered Learning (LL), the adopted classifier in each layer was a Light Gradient Boosting Machine	60 min	60 min	MIMIC III (15)	Accuracy (%) = 75.9 ± 4.2
Tang et al. (16)	30	N/R	Patient's NE infusion rate per unit weight, ECG, ABP	125 Hz	HR, PP, KR	•Physiology based approach (our method) •RRLS •IIR filter •ARMAX	1.3–6.67 h	3.33–20 min	Inter-mountain Medical Center, MIMIC-III (15)	Mins to 5 mmHg RMSE •Our method = 11.5 •RRLS = 9.5 •IIR = 8 •ARMAX <3
Lee et al. (17)	3,301	*F* = 1,479	•Invasive: AP+ECG +PPG+EtCO2 vs. AP •Non invasive: ECG+ PPG+EtCO2 vs. PPG	100 Hz	N/R	DL consisted of seven convolutional layers	30 s	5, 10, 15 min	VitalDB database (18)	AUROC(%) •Invasive: 89.7 vs. 89.1 •Non invasive: 76.2 vs. 69.4
Moghadam et al. (19)	1,000	*F* = 396	ABP, HR, SBP, DBP, Resp, SpO2; PP, MAP, CO, MAP2HR, RR	N/R	33 scalar feature	•LR •SVM •KNN •Decision tree •Discriminant analysis •Naive Bayes •Ensemble	5 min	30 min	MIMIC III (15)	Accuracy(%) •LR = 95 •SVM = 94 •KNN = 92 •DT = 93 •DA = 93 •NB = 88 •Ensemble = 93
Lee et al. (20)	282	*F* = 148	Non invasive BP, HR, Mechanical Ventilation data, Bispectral index	N/R	Min, max, mean, std. Experiment performed 3-fold: 27, 56, 67 features then sum and dim reduction to get 98, 45, 20, 29. Best performance are 56 for 1st experiment and 20 for 2nd experiment	•RF •Xgboost •CNN	4–1 min before intubation	1 min	Soonchunhyang University Bucheon Hospital database	Accuracy (%) Raw feature vs. plus statistics features: •RF =70.3 vs. 74.9 •CNN = 72.6 vs. 69.0 •Xgboost = 64.6
Moghadam et al. (21)	1,000	N/R	ABP,HR,SBP, DBP, Resp, SpO2; PP, MAP, CO, MAP2HR, RR	N/R	33 scalar features. PCA optimize the feature set and resulted into 11 combined features	•LR •SVM •KNN •DT •Discriminant analysis •Naive Bayes •Ensemble	5 min	30 min	MIMIC III (15)	Accuracy(%) •LR = 95 •SVM = 94 •KNN = 92 •DT = 93 •DA = 93 •NB = 88 •Ensemble = 93
Xiao et al. (22)	2,866	N/R	ABP	N/R	decompose MAP with SW and ensemble EMD, then a 3-layer auto encoder to get 50 outputs	Multiple gene expression programming classier	2 h	60 min	MIMIC II (13)	Voting combination strategy vs. 10-fold cross validation (%) •Accuracy = 85.7 vs. 86.2 •Sensitivity = 86.4 vs. 86.8 •Specificity = 85.5 vs. 85.9
Shin et al. (23)	207	N/R	MAP	N/R	•LR: mean, slope, SD of MAP of past 5, 10, 20, 30, 45, 60 min •AR: MAP values with *t* and preceding 5 min time-steps were computed as the median MAP	•Logistic regression (LR) •Auto-regressive model (AR)	60 min	30 min	MIMIC II (“Hospital 1”) (13), Mass General Hospital (“Hospital 2”)	•LR predicted on average 7.0 min before onset (Hospital 1) and 2.5 min before (Hospital 2) •AR predicted 10.5 and 2.0 min before
Chan et al. (24)	538	N/R	MAP, HR, SPO2	N/R	N/R	Long Short-Term Memory, three layers each with 100 units	10–60 min	10–60 min	Kingston General Hospital	•Accuracy(%) = 80 •AUC(%) = 87
Angelotti et al. (25)	86	N/R	ABP and ECG containing at least one ECG lead	N/R	SBP statistical moments; LF, HF, VLF spectral powers (for both RR and SBP); LF/HF (for both RR and SBP); Baroreflex amplitude; Baroreflex frequency	•4 classification trees •6 SVM •6 KNN •LR	20 min	10 min	MIMIC III (15)	AUC(%) with vs. w/o BRFX: •Trees = 67 vs. 63 •SVM = 68 vs. 62 •KNN = 64 vs. 57 •LR = 62 vs. 54
Pathinaru-pothi et al. (26)	30	N/R	MAP	N/R	Use MAP severity quantizer, Consensus motif extractor, SVM based prediction engine for feature extraction	SVM	15 min	2.75 h	MIMIC II (13)	F1 score (%) = 82
Kim et al. (27)	2,291	N/R	MAP	N/R	N/R	Collision Frequency Locality Sensitive Hashing	5 h	60 min	MIMIC II (13)	Accuracy (%) in the range (93, 96)
Hamano et al. (28)	100	N/R	MAP, EtCO2, MAC, HR, SpO2, and body temperature	N/R	Each variable is mapped *via* the similarity-based approach, and trial and error to get 6,000 combinations	Spiking neural networks	15 min	5 min	OR of a tertiary hospital, Auckland NZ	37.6% of the experiments had an SNR above 0, which means better prediction than the naive method
Jiang et al. (29)	2,866	N/R	MAP	1 Hz	55 feature incl peak, mode, skewness, kurtosis, and Shannon entropy from original time series, first 9 IMFs and last IMF	Multi GP	2 h	60 min	MIMIC II (13)	Accuracy (%) = 82.9 in the training set and 79.9 in the testing set
Ghosh et al. (30)	50	N/R	MAP	N/R	A gap-constrained sequential contrast pattern P is required (1) Positive Support: countP (D+, g) >= alpha (2) Negative Support: countP (D-, g) < = delta	Sequential pattern mining	30, 60 min	60, 120 min	MIMIC II (13)	Accuracy (%) single mode performance with 10 symbols vs. multi mode performance with 15 symbols: •30 min = 82.3 vs. 81.3 •60 min = 83.6 vs. 80.9
Ghosh et al. (31)	528	N/R	MAP	N/R	Utilize sequential contrast patterns as features to build classification models	SVM	60, 90 min	30, 60 min	MIMIC II (13)	Accuracy (%) = 85.8
Kim et al. (32)	2,291	N/R	MAP	N/R	N/R	LSH with two variants, the bit sampling based (L1LSH), the random projection based (E2LSH)	5 h	60 min	MIMIC II (13)	Accuracy (%) •L1LSH >95 •E2LSH >90
Jiang et al. (33)	2,866	N/R	MAP	1 Hz	EMD to decompose MAP into 77 IMFs. Statistical features: min, mean, max, median, variance, max instantaneous freq, HF/LF energy ration	•Multi GP •SVM	2 h	60 min	MIMIC II (13)	Accuracy (%) •MGP = 79.1 in training set and 78.0 in testing set •SVM = 76.2 and 75.5
Fan et al. (34)	1,599	N/R	ABP	125 Hz	EMD to extract 77 features then group to 30. Extracted features: min,mean,max, median,variance. Calculated features: max instantaneous freq, HF/LF. Also, the 12th percentile, skewness, kurtosis, mode of the last IMF	RF based on GP	30 min	N/R	MIMIC II (13)	Accuracy (%) = 77.6
Kim et al. (35)	2,291	N/R	ABP	125 Hz	The first and second differences, 20-min variance and slope	•Dynamic Bayesian network •KNN	30 min	30, 60 min	MIMIC II(13)	Accuracy (%) •DBN = 80 •KNN = 82
Jiang et al. (36)	110	N/R	MAP	N/R	EMD was used to calculate MAP time series and BW of the AM, FM, power of IMF	GP	2 h	60 min	MIMIC II (13)	Accuracy (%) = 83.4 in the training set and 80.6 in the testing set
Zhang et al. (37)	12	N/R	MAP, HR, SBP, and DBP	N/R	MAP, HR, SBP, and DBP	ANN with one hidden layer	30 min	60 min	MIMIC II (13)	Median Absolute Difference between the predicted and actual HI was 0.070, ranged from 0.012 to 0.175
Sun et al. (38)	2,863	N/R	MAP	1 Hz	The 2 cluster centers, x1Mean and x2Mean, the 2 cluster ratios, x1Ratio and x2Ratio, the average of 15 min MAP signal before T0	SVM	60 min	60 min	MIMIC II (13)	•Accuracy = 81.2% •Sensitivity = 83.2% •Specificity = 80.4%
Janghorbani et al. (39)	95	N/R	HR, SAP, DAP, MAP	N/R	•LR: 10% DAP, mean MAP, max ECO; •LR+GA: same as LR, 95% ECO, skewness dHR, mean ECO slope, 5% ECO slope, mean MAP slope, mean DAP slope; •SVM+GA: HR/SAP IPR 10-5%, 50% HR/MAP, 95% dHR, skewness dHR, mean MAP slope, 5% MAP slope, 5% SAP slope;	•LR •SVM	30 min	60 min	MIMIC II (13)	Accuracy (%) •LR = 80 •LR+GA = 86 •SVM+GA = 88
Rocha et al. (40)	311	N/R	MAP	125 Hz	N/R	Neural network multi-models	12 h	60 min	MIMIC II (13)	•Sensitivity = 82.8% •Specificity = 78.4%
Sun et al. (41)	1,500	N/R	SBP, DBP, MAP, SpO2, HR	N/R	top-10 wavelet coefficients as the features	Locally Supervised Metric Learning (LSML)	60 min	60 min	MIMIC II (13)	Accuracy (%) = 85.51
Lee et al. (42)	1,357	N/R	HR, SBP, DBP, MAP	N/R	Mean, median, SD, variance, interquartile range, skewness, kurtosis, linear regression slope, and relative energies in different spectral bands. A total of 45 features, whose space dim was reduced *via* PCA to 15–16	Feed-forward, three-layer artificial neural networks (ANNs)	30 min	1–4 h	MIMIC II(13)	Accuracy (%) •1 h = 87.3 ± 0.8 •2 h = 84.2 ± 1.4 •3 h = 83.5 ± 1.7 •4 h = 81.0 ± 1.9
Afsar (43)	60	N/R	ABP	125 Hz	SBP and Area under SBP wave along with the 1st, 3rd, and 6th principle component averaged over beats in each 60 s interval	Linear support vector machine (LSVC)	1.5 h	60 min	MIMIC II (13)	Accuracy (%) •No Feature Reduction = 79.4 •Using GA Features = 93.7
Wang et al. (44)	70	N/R	MAP	N/R	db3 as wavelet mother function to decompose the MAP signal at three levels to get the LF coefficient cA3 and HF coefficients cD1, cD2, and cD3. Then extract median and maximum	SVM	60 min	60 min	MIMIC II (13)	Accuracy (%) = 90
Rocha et al. (45)	110	N/R	ABP	N/R	The filtered signals are down-sampled by 2 and the results are called approximation and detail coefficients	Feed-forward neural Networks with two hidden layers	2 h	60 min	MIMIC II (13)	•Sensitivity = 94.7% •Specificity= 93.6% •Accuracy = 94.0%
Fournier et al. (46)	60	N/R	ECG, PAP, ABP, central venous pressure, HR, RR, SpO2, CO, and alarms annotations	N/R	Use KL divergence to identify the most discriminative features	Nearest neighbors (NN)	30 min		MIMIC II (13)	Accuracy (%) = 80
Jousset et al. (47)	50	N/R	MAP	N/R	N/R	SVM	2 h	60 min	MIMIC II (13)	Accuracy (%) = 75
Chiarugi et al. (48)	60	N/R	ECG,ABP	125 Hz	HR, SBP, mean ABP (ABPM), DBP, MAP	Decision tree	10 h	2 h	MIMIC II (13)	Accuracy (%) 91.7 in the training set and 75 on test set
Henriques et al. (49)	50	N/R	ABP	N/R	N/R	Generalized regression neural network	6 h	60 min	MIMIC II (13)	Accuracy (%) 100 for test set A, 92.5 for test set B

Overview of the included papers in this study. STD/SD, standard deviation; LR, logistic regression; SVM, support vector machine; RF, random forest; ABP, arterial blood pressure; HR, heart rate; SBP, systolic blood pressure; DBP, diastolic blood pressure; MAP, mean arterial pressure; PP, pulse pressure; CO, cardiac output; NE, norepinephrine; KNN, K-nearest neighbors; SW, sliding window; EMD, empirical mode decomposition; GP, genetic programming; IMF, intrinsic mode functions; GA, genetic algorithm.

**Figure 2:**
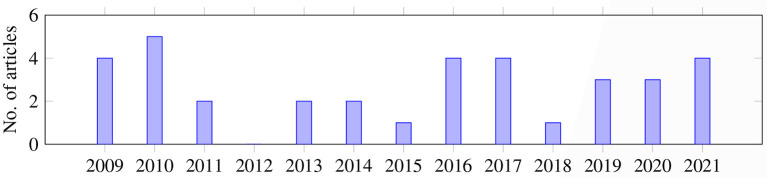
Number of publications by year.

Overall, as shown in Figure 2, the articles were published during the years of the search range; most of the articles were published in 2010 (*n* = 5), followed by 2009, 2016, 2017, 2021 (*n* = 4, each). In 2019 and 2020, three articles were published each year. In 2011, 2013, and 2014, only two papers were published on this topic. In 2015 and 2018, only one article was published each year.

### Results of studies

Most of the studies (37%, 13 out of 35) were large-scaled (1,110–4,000 patients were included). However, small-sized studies were also common: seven studies included 60–100 patients, six studies included 12–50 patients, and five studies included 110–500 patients. Medium-sized studies were relatively rare; only three studies had 510–1,000 patients. Of the 35 articles, 31 did not report the gender of the subjects; of those that did report on gender, the percentage of females was 40% (*n* = 2), 44.8% (*n* = 1), and 52.5% (*n* = 1). More than half of the articles (*n* = 18) used the arterial blood pressure (ABP) signal to focus on MAP, while some (*n* = 10) used more than three data sources. A few studies (*n* = 7) used ABP+ECG as the signal inputs. The majority of the articles (*n* = 25) did not mention what sampling frequency was used to acquire the signals. Of those that did report on this factor, 125 Hz was most frequently used (*n* = 6), followed by 1 Hz (*n* = 3) and 100 Hz (*n* = 1). Some (*n* = 8) of the 35 articles did not mention the features extracted because they simply used the raw data. Five articles described the methodology used to extract the features, but did not mention the exact number of features. Seven articles described the methodology and provided the final number of features, but they did not mention what the features were. Fifteen articles provided a list of the features that were extracted.

Statistics of the raw signal (e.g., maximum, minimum, mean) were the most common features extracted; this was adopted by 13 out of the 27 studies that described the methodology to extract the features, followed by clinical equations that were calculated based on the raw signal (*n* = 7), Empirical Mode Decomposition (*n* = 5), wavelet transform (*n* =3), and contrast sequential pattern and sliding window (*n* = 2 each). Only five of the 27 studies also conducted feature reduction, and Principal Component Analysis was the only methodology used by more than one study (*n* = 2).

Most of the studies (*n* = 24) only investigated one machine learning algorithm. A few of the studies (*n* = 6) evaluated three to seven machine learning algorithms and a few (*n* = 5) compared two machine learning algorithms. Of the type of machine learning algorithms used, Support Vector Machine (SVM) was the most studied (*n* = 12), followed by Logistic Regression (LR) and Artificial Neural Network (ANN) (*n* = 6 each). Other common machine learning algorithms include Nearest Neighbors (KNN) (*n* = 5), Genetic Programming (GP) (*n* = 4), Random Forest (RF), Gradient Boosting Machine, Decision Tree, Naive Bayes (NB), or Dynamic Bayesian Network (*n* = 3, each), and Locality Sensitive Hashing (LSH) (*n* = 2). The least examined algorithms were Deep Learning, Spiking Neural Network, Sequential Pattern Mining, and Long-Short-Term Memory (*n* = 1, each).

Most of the articles (*n* = 27) reported accuracy as the evaluation metric for the machine learning algorithm(s) that were studied. Eight other articles had their own way of measuring performance without assessing accuracy; those methods included F1 score, area under the curve (AUC), sensitivity (SE), and specificity (SP), prediction time, and the absolute difference between the prediction and the actual hypotension index.

The length of the prediction window depends on the time the AHE is expected to happen. Among the 35 articles, most of the studies (*n* = 30) focused on predicting AHE in the ICU, where the most common prediction window was 60 min prior to the onset of the event (*n* = 21), followed by 30 min (*n* = 4), 120 min (*n* = 2), or 165 and 10 min (*n* = 1, each). Some articles looked at a time range, for example 10–60 min or 1–4 h (*n* = 1, each). Two studies did not report prediction window, assuming that the prediction window occurred right after the observation window.

A second type of prediction looked at intra-operative AHE; but only three studies focused on this area. Therefore, the prediction window is very short, either 1 min (*n* = 1), 5 min (*n* = 1) or 5, 10, or 15 min (*n* = 1), because intra-operative AHE occurs during anesthesia and only after intubation. The last type of study checked AHE that occurred during medication against septic shock. Data from patients given vasopressor infusion (*n* = 1) or norepinephrine infusion (*n* = 1) were studied to predict AHE. The prediction window for this type of forecast was 30 min (*n* = 1) or 3–20 min (*n* = 1) before the onset of the AHE.

Similarly, the observation window depends on whether the AHE is post-operation, intra-operative, or occurs when taking medication. For the first type of AHE, the observation window values, ranging from most common to least common, are 30 min (*n* = 7), 60 min (*n* = 6), 2 h (*n* = 6), 5 h (*n* = 3), 5 min (*n* = 2), 90 min (*n* = 2), 15 min (*n* = 1), 20 min (*n* = 1), 10 h (*n* = 1), 12 h (*n* = 1), 6 h (*n* = 1), or 10–60 min (*n* = 1). For intra-operative AHE prediction, the observation windows are 1–4 min before intubation, 30 s and 15 min (*n* = 1, each). For the AHE prediction during medication, the observation window is either 60 min or 1.3–6.67 h (*n* = 1, each).

The MIMIC-II database (*n* = 26) was the most frequently used data source, followed by the MIMIC-III database (*n* = 4), or a hospital database that is not public (*n* = 3). The Vital DB was rarely used (*n* = 1); it is a public database. One study used both the MIMIC-II database and a hospital database.

## Discussion

The sample sizes of the studies varied greatly, ranging from a very small-scaled analysis with only a few dozen patients to very large-scaled studies that include several thousand people. Such a large variation in the number of subjects means that a comparison of different studies is not possible or could be strongly biased. Kim et al. (35) demonstrated that the performance of both of the chosen algorithms improved up to a point when the size of the training dataset increased. Patient information, including gender, age, comorbidity, medication, etc., was not reported in most of the reviewed studies, and these factors could have an impact on whether an AHE could occur.

The MIMIC database was mostly often used in the reviewed studies, probably due to its freely accessible nature. There might be data quality concerns regarding this database, for example, missing data. Moreover, the database is continuously updated, meaning that different studies, although all referring to the same database, might not have used the same data.

**Figure 3:**
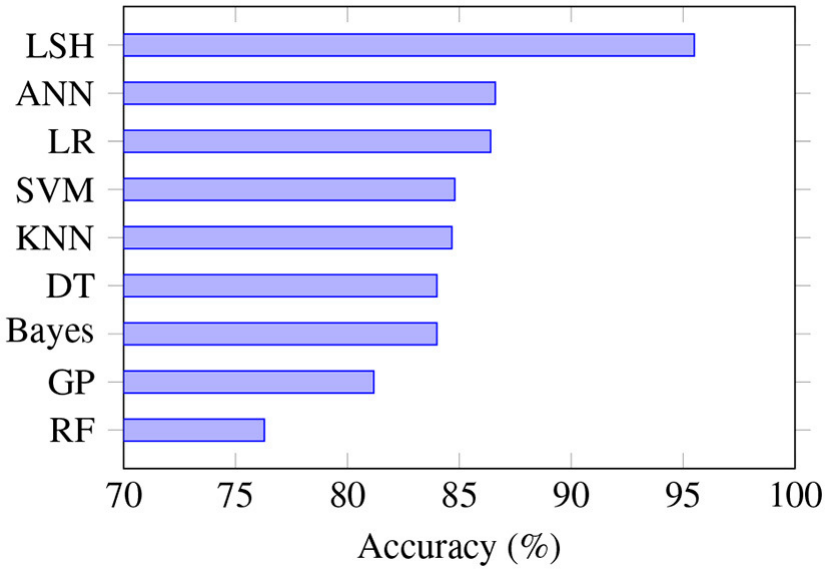
Prediction accuracy based on the type of algorithm. LSH is the most accurate algorithm, although only a few studies used it. SVM is the algorithm that was most often studied, and RF is the least accurate algorithm. RF, random forest; GP, genetic programming; DT, decision tree; KNN, K-nearest neighbors; SVM, support vector machine; LR, logistic regression; ANN, artificial neural network; LSH, locality sensitive hashing.

Since accuracy was the performance measure mostly often reported, we compared the performance of different machine learning algorithms based on this evaluation metric (Figure 3). LSH has the highest average accuracy; however, only two studies used this algorithm, and both had the same first author: Kim and O'Reilly (32). In contrast to some other algorithms that are better established and more widely studied, the performance of LSH needs to be further assessed in future studies. Kim and O'Reilly (32) observed that LSH variants have very different robustness against data irregularities, and noted that further work is needed to develop an effective data representation that can be integrated into the general LSH framework.

### AHE prediction

When researchers use the same data but different prediction window, as shown in Table 2, they will get different results even with the same machine learning algorithm. In our analysis, we found there is no standard prediction window consensus in this area yet. Thus, the choice of prediction window is mostly subjective. Zhang et al. (12) analyzed the impact of prediction gaps on six machine learning algorithms and concluded that some methodologies are less impacted than others when the prediction gaps change. Lee et al. (42) studied the gap window size ranging from 1 to 4 h and showed that, in general, the overall performance degrades as the gap size increases.

**Table 2:** Summary of the prediction window.

**Type**	**Time length (mins)**	**Number of study**
Post-operative AHE	10	1
	30	4
	60	21
	120	2
	165	1
Intra-operative AHE	1	1
	5	2
	10	1
	15	1
AHE during medication	3–20	1
	30	1

Most studies focused on predicting post-operative AHE. A 60 min prediction time length was the most common. Fewer studies aimed to predict AHE during an operation or when taking medication.

Regarding the training data time length, Lee et al. (20) studied the intra-operative AHE scenario and found 3 min of data performed better than 2 and 1 min. It is not difficult to imagine that a shorter prediction window and a longer training data time would provide better prediction accuracy, but a prediction window that is too short would be clinically less valuable to healthcare providers in terms of providing them with sufficient time to check the patient's situation and decide if an intervention is needed.

Summarization frequency may also impact the accuracy performance. In Pathinarupothi et al. (26) summarization was done once every 5 and 10 min; they found that a 10 min summarization can predict AHE with at least a 10% better F1 score, on average.

### Feature extraction

Feature selection is the process of trying to fit the dataset. As mentioned in the previous three sub-sections, the missing patient background information, the diversity in the sample size and the prediction window, and the dynamics of the database can impact the data to be studied, while directly impacting the features to be selected.

**Figure 4:**
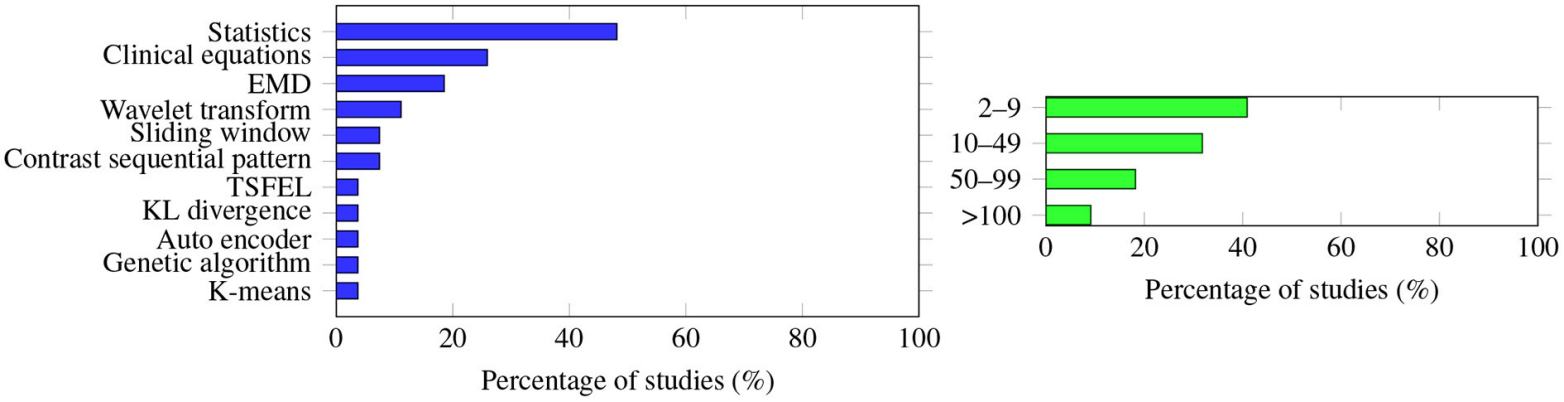
**(Left)** The methodology used to extract the features was very diverse; no single methodology accounts for more than half of the studies. Statistics (e.g., maximum, minimum, mean values of the raw data) are the extracted features most often studied, followed by clinical equations (apply raw data to the equation to calculate some of the derived information, e.g., cardiac output, MAP to HR ratio). **(Right)** Graph showing how many features were extracted to predict AHE. The number varies greatly among the studies, with a single feature extraction being the most common.

However, the way in which the features were extracted also varied in the studies. While many studies described what methodology was used to extract the features (27 out of 35 articles), as shown in Figure 4 (left panel), eight articles did not provide details, mostly because raw data were applied and not processed. Kim et al. (35) found that the performance of the models utilizing derived features was worse than the performance of the models simply using the raw time series. In the study of Zhang et al. (12) feature reduction did not impact the accuracy or AUC performance of the selected machine learning algorithms. Afsar (43) reported that using dimensionality reduction effectively improved the prediction accuracy, and only five features were needed for the calculation. Note that the most used number of features is between 2 and 9, as shown in Figure 4 (right panel). Lee et al. (20) compared the use of vital records with the use of vital records plus electronic health records (EHR), and found that for the convolutional neural network model, EHR improves the accuracy by 0.39%; however, for other algorithms, such as RF, Xgboost, and deep neural network, the differences were negligible. Therefore, with these completely different findings, it is difficult to conclude which methodology is the best for extracting the features, what features are universally effective no matter what algorithms are applied, or how feature reduction impacts prediction performance.

When considering the different combinations of feature extractions and time windows, the situation could become very complicated. Ghosh et al. (30) studied different combinations of observation windows (30, 60 min), prediction windows (60, 120 min), and feature classification methods (single mode, multi-mode). They found that the prediction accuracy was the highest when both the observation window and prediction window times were 60 min for a single mode extraction mechanism, but the highest prediction accuracy occurred when the observation window was 30 min and the prediction window was 60 min for the multi-mode classification method.

### Evaluation metrics

**Figure 5:**
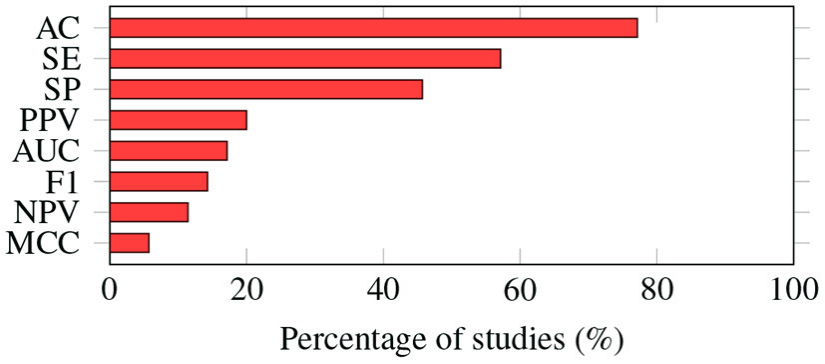
Evaluation metrics. The way in which the performance of machine learning algorithms is evaluated varies from study to study. Accuracy is the most common metric; it was adopted by almost 80% of the studies. AC, accuracy; SE, sensitivity; SP, specificity; PPV, positive predictive value; AUC, area under the ROC curve; F1, F1-score; NPV, negative predictive value; MCC, Matthews correlation coefficient.

As shown in Figure 5, most of the studies (*n* = 27) reported accuracy as one of the evaluation metrics, followed by sensitivity (*n* = 17) and specificity (*n* = 16).

However, Ribeiro et al. (14) and Moghadam et al. (21) mentioned that the common ways of measuring performance (including accuracy, sensitivity, specificity, etc.) might not be sufficient to evaluate the performance of an algorithm in predicting AHE, as the data are highly skewed. In Moghadam et al. (21) the Naive Bayes (NB) algorithm raised 17,271 false positive alarms; true positive was only seen in 23,552 cases. However, the accuracy, sensitivity, and specificity of NB were 88, 85, and 88%, respectively. Based on those results, NB is a good machine learning algorithm candidate. However, in clinical practice, such a high false alarm rate means the healthcare providers would gradually lose confidence in the accuracy of the alarm and may not react when a true positive incident occurs. Both authors suggested using positive predictive value (PPV) or the F1 score to evaluate the machine learning algorithm to predict AHE, but PPV and F1 scores are missing in many of the current studies.

Deep learning models have generated great interest due to breakthroughs in fields like image analysis and speech recognition. However, we noticed that as regards to predicting AHE, deep learning algorithms were not so widely studied, and the performances were not better than other traditional methods (17, 24, 37). One possible explanation could be that deep learning models require a massive dataset for learning, which is usually lacking in the ICU setting; therefore, the interest in exploring deep learning's potential in predicting AHE is less prominent. Another reason might be that deep learning is known to be good at learning from features. At the same time, some research (35) has shown that for predicting AHE, raw data could sometimes be even better, probably because MAP itself is already a good indicator. Thus, a simpler but faster model could be sufficient to fulfill the expectation in AHE prediction.

### Recommendation for future work

Summarizing the study findings, we recommend that researchers consider the following aspects when designing future studies:

1. Use a large number of subjects (>100) balanced in gender, age, and ethnicity. Moreover, the health status of the subjects needs to be stated, such as comorbidities.
2. Examine a consistent prediction window, precisely 30, 60 min, or both.
3. Elaborate on the feature selection phase, including the number of features extracted, how the feature extraction was done, and what the features are, since these aspects would impact the algorithm performances.
4. Report different evaluation metrics such as accuracy, sensitivity, specificity, Matthews correlation coefficient (MCC), and F1 score is essential for objective assessment.

### Limitations

Due to time constraints, we searched only four databases. It is, therefore, possible that we missed some articles available in other databases. The keyword choice might also lead to the omission of relevant research. Some studies checked applications of machine learning algorithms in multiple areas, which could include but are not limited to AHE prediction.

## Conclusion

This review summarizes the application of machine learning algorithms for predicting AHE in articles published from 2008 to 2021. Most of the studies included in the review focused on the prediction of post-operative AHE 30 or 60 min before the onset utilizing ABP signals from the MIMIC database. The machine learning algorithm showed an accuracy between 76.3 and 96.5%. The machine learning algorithms perform well when evaluated with metrics like accuracy, sensitivity, and specificity. However, some researchers (14, 21) reported high false positives in some algorithms, when using metrics like PPV or F1 score. As many of the studies currently do not report MCC or F1 score, it is difficult to say if and which of the machine learning algorithms are ready to be used clinically.

By examining the metrics and machine learning algorithms used in previous studies, this review aimed to enable future researchers to better design experiments and pave the way for the findings to be adopted in a clinical environment. Little evidence is currently available for a meta-analysis due to the variations in the scope and methodologies used in previous studies. Further research is needed to evaluate the technology in real life and examine its impact on patients and healthcare providers.

## Data availability statement

The original contributions presented in the study are included in the article/Supplementary material, further inquiries can be directed to the corresponding author.

## Author contributions

ME designed and led the study. AZ, ME, and CM conceived the study. All authors approved the final manuscript.

## Funding

This work was supported by open access funding provided by ETH Zurich.

## Conflict of interest

The authors declare that the research was conducted in the absence of any commercial or financial relationships that could be construed as a potential conflict of interest.

## Publisher's note

All claims expressed in this article are solely those of the authors and do not necessarily represent those of their affiliated organizations, or those of the publisher, the editors and the reviewers. Any product that may be evaluated in this article, or claim that may be made by its manufacturer, is not guaranteed or endorsed by the publisher.
