# Supplementary material for: Machine learning for predicting acute hypotension: A systematic review
Source: Front Cardiovasc Med. 2022 Aug 23;9:937637. doi: 10.3389/fcvm.2022.937637 (PMC9445248; doi:10.3389/fcvm.2022.937637)
Supplement: Supplementary file 1 [file Data_Sheet_1.docx]

The PubMed, IEEE database, Embase, and Google Scholar were searched for articles published between January 1, 2008, and January 1, 2022, for all English-language papers using the following Medical Subject Headings (MeSH):

("antihypertensive agents"[Pharmacological Action] OR "antihypertensive agents"[MeSH Terms] OR ("antihypertensive"[All Fields] AND "agents"[All Fields]) OR "antihypertensive agents"[All Fields] OR "hypotensives"[All Fields] OR "hypotension"[MeSH Terms] OR "hypotension"[All Fields] OR "hypotensions"[All Fields] OR "hypotensive"[All Fields] OR ("antihypertensive agents"[Pharmacological Action] OR "antihypertensive agents"[MeSH Terms] OR ("antihypertensive"[All Fields] AND "agents"[All Fields]) OR "antihypertensive agents"[All Fields] OR "hypotensives"[All Fields] OR "hypotension"[MeSH Terms] OR "hypotension"[All Fields] OR "hypotensions"[All Fields] OR "hypotensive"[All Fields]) OR ("hypotension"[MeSH Terms] OR "hypotension"[All Fields] OR ("low"[All Fields] AND "blood"[All Fields] AND "pressure"[All Fields]) OR "low blood pressure"[All Fields])) AND ("electrocardiography"[MeSH Terms] OR "electrocardiography"[All Fields] OR "ecg"[All Fields] OR ("electrocardiogram s"[All Fields] OR "electrocardiography"[MeSH Terms] OR "electrocardiography"[All Fields] OR "electrocardiogram"[All Fields] OR "electrocardiograms"[All Fields]) OR ("mimic"[All Fields] OR "mimics"[All Fields])) AND ((("automatable"[All Fields] OR "automatic"[All Fields] OR "automatical"[All Fields] OR "automatically"[All Fields] OR "automaticities"[All Fields] OR "automaticity"[All Fields] OR "automatics"[All Fields] OR "automatism"[MeSH Terms] OR "automatism"[All Fields] OR "automatisms"[All Fields] OR "automatization"[All Fields] OR "automatize"[All Fields] OR "automatized"[All Fields] OR "automatizes"[All Fields] OR "automatizing"[All Fields]) AND ("detect"[All Fields] OR "detectabilities"[All Fields] OR "detectability"[All Fields] OR "detectable"[All Fields] OR "detectables"[All Fields] OR "detectably"[All Fields] OR "detected"[All Fields] OR "detectible"[All Fields] OR "detecting"[All Fields] OR "detection"[All Fields] OR "detections"[All Fields] OR "detects"[All Fields])) OR ("machine learning"[MeSH Terms] OR ("machine"[All Fields] AND "learning"[All Fields]) OR "machine learning"[All Fields]) OR ("artificial intelligence"[MeSH Terms] OR ("artificial"[All Fields] AND "intelligence"[All Fields]) OR "artificial intelligence"[All Fields]) OR ("deep learning"[MeSH Terms] OR ("deep"[All Fields] AND "learning"[All Fields]) OR "deep learning"[All Fields]) OR ("predict"[All Fields] OR "predictabilities"[All Fields] OR "predictability"[All Fields] OR "predictable"[All Fields] OR "predictably"[All Fields] OR "predicted"[All Fields] OR "predicting"[All Fields] OR "prediction"[All Fields] OR "predictions"[All Fields] OR "predictive"[All Fields] OR "predictively"[All Fields] OR "predictiveness"[All Fields] OR "predictives"[All Fields] OR "predictivities"[All Fields] OR "predictivity"[All Fields] OR "predicts"[All Fields]))
